# Progression of fertility-sparing treatment for atypical endometrial hyperplasia in a woman with lynch syndrome: a case report and review of the literature

**DOI:** 10.3389/fonc.2024.1422006

**Published:** 2024-08-12

**Authors:** Ya-Ting Hsu, Chi-Hau Chen

**Affiliations:** Department of Obstetrics and Gynecology, National Taiwan University Hospital, Taipei, Taiwan

**Keywords:** lynch syndrome, fertility-sparing treatment, atypical endometrial hyperplasia, endometrial cancer, oncology outcome

## Abstract

Endometrial cancer in Lynch syndrome is characterized by a higher incidence, younger age at onset, and increased recurrence rates compared to sporadic cases, while the safety and efficacy of fertility-sparing treatments remain uncertain. This case report presents the oncology outcome of fertility-preserving treatment in a 39-year-old woman diagnosed with Lynch syndrome and atypical endometrial hyperplasia. Initially, she responded favorably to fertility-preserving treatment but subsequently experienced disease relapse and rapid progression during retreatment. Final pathology revealed endometrial cancer with metastasis to the right ovary, categorized as FIGO 2023 stage IIIA1. This population’s unique molecular mechanisms and genetic mutations warrant special consideration when opting for fertility-sparing treatment. We have reviewed and summarized the oncology and pregnancy outcomes among Lynch syndrome and MMR-deficient patients through previous literature. However, no studies have investigated retreatment after recurrence in Lynch syndrome. Our case highlights the potential risks associated with retreatment following relapse. Vigilant monitoring and prompt consideration of surgical intervention are recommended upon disease relapse.

## 1 Introduction

Endometrial cancer (EC) is one of the most common types of gynecologic cancers found worldwide. While more frequently diagnosed in postmenopausal women, 3.1-8% of endometrial cancer cases occur below the age of 40 in Taiwan (1, 2). The standard treatment for early-stage EC and atypical endometrial hyperplasia (AEH) involves definitive surgeries. However, for young women desiring fertility preservation, conservative treatment becomes an important issue. Hormonal therapy, including oral or intrauterine progestins, followed by endometrial biopsies every 3-6 months, is recognized as an alternative for those seeking to maintain fertility. The outcomes of fertility-sparing treatment in EC and AEH generally yield favorable results. The complete response (CR) rate and recurrence rate vary among different studies, ranging from approximately 66% to 76.3% and 20.1% to 30.7%, respectively (3–5).

Lynch syndrome (LS) is a hereditary cancer syndrome caused by germline mutations in mismatch repair (MMR) genes, including MLH1, MSH2, MSH6, and PMS2. These genes are responsible for correcting errors acquired during DNA synthesis. Loss of function in these genes markedly increases the risk of developing cancer. Approximately 1.8-2% of ECs can be attributed to LS (6). The onset of EC in LS occurs at a younger age, typically ten years earlier than in the general population, with an average age of 49 years (7). Gynecologic cancer often acts as sentinel cancer, preceding the onset of colon cancer in half of the LS cases (8). Given the comparatively younger age at diagnosis, fertility-sparing treatment becomes particularly crucial for this patient group. However, the safety and effectiveness of fertility-preserving treatment in LS patients remain uncertain. We herein present a case of AEH in a patient with LS receiving fertility-sparing treatment at our institution. This patient experienced two recurrences after achieving CR and underwent a third course of medication treatment. Unfortunately, her disease rapidly progressed to FIGO (2023) stage IIIA1 endometrial cancer. This study highlights the unique nature of LS-associated EC and the considerations before opting for fertility-sparing treatment.

## 2 Case presentation

A 39-year-old nulliparous woman presented to our institution for an infertility assessment. Her medical history is significant for LS, which was confirmed through genetic testing at the age of 8. Notably, her paternal family history is marked by multiple malignancies. Her father was diagnosed with colon adenocarcinoma at age 55 and gastric cancer at age 56. Additionally, her grandmother was diagnosed with gastric cancer in her 50s. Her paternal aunts have also been diagnosed with colon cancer, pancreatic cancer, and endometrial cancer, all linked to LS.

Upon evaluation at our clinic, a transvaginal ultrasound revealed an endometrial lesion, which was later confirmed by pathology as atypical complex hyperplasia. She denied experiencing abnormal vaginal bleeding before the biopsy. Following a thorough discussion with the patient, she opted for fertility-preserving treatment. The patient achieved CR after three months of oral progestin therapy with 125 mg medroxyprogesterone acetate (MPA). However, a recurrence of atypical complex hyperplasia was confirmed by hysteroscopic endometrial lesion resection 12 months later. After six months of medical treatment with MPA 250 mg, a second CR was attained. Subsequently, the patient underwent infertility treatment, during which persistent vaginal bleeding was noted. Hysteroscope demonstrated small foci of grade 1 endometrioid adenocarcinoma. The recurrence interval from the second CR was approximately four months.

**Figure 1:**
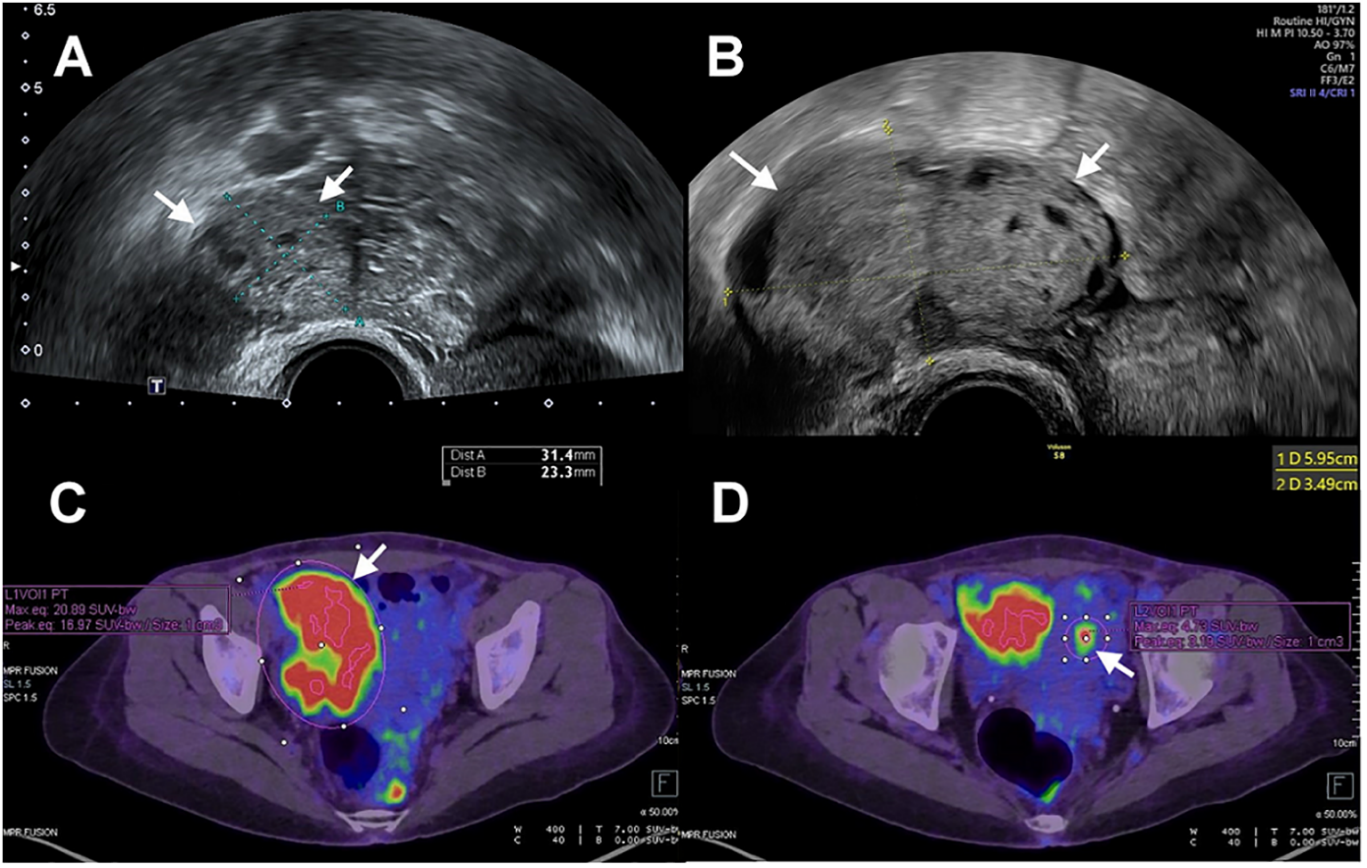
**(A)** Transvaginal ultrasonography showed a right ovary (arrow) measuring 3 cm in size. **(B)** The size of the right ovary (arrow) increased from 3cm to 6 cm with a three-and-a-half-month interval between examinations. **(C)** PET scan showed increased FDG accumulation of right ovary (arrow). **(D)** PET scan showed increased FDG accumulation of uterine cavity (arrow).

Magnetic resonance imaging (MRI) examination reported the lesion limited to the endometrium, with the tumor marker CA-125 value within normal limits. Despite the recommendation for surgery, the patient chose to continue medication to preserve fertility. After three months of MPA 500 mg per day, she presented with a partial response (PR) with pathology-confirmed atypical hyperplasia. Then, the treatment regimen was transitioned to Megestrol acetate at 320 mg per day for an additional three months. During a routine ultrasound examination, a newly developed right ovarian tumor measuring 5.9 x 3.5 cm was detected, which had not been identified three and a half months earlier (**Figures 1A, B**). The tumor exhibited prominent blood flow on Doppler imaging. Meanwhile, the endometrium thickness was within the normal range (1.5 mm). The CA-125 level showed a sudden elevation (287.6 U/mL). Consequently, the patient decided to undergo surgery. Preoperative computed tomography (CT) imaging demonstrated a 7.1 cm right ovarian tumor without evidence of endometrial lesions or lymphadenopathy. The whole-body positron emission tomography (PET) result was consistent with right adnexal malignancy (SUVmax.eq =20.9; **Figure 1C**). Focal FDG accumulation in the uterine cavity (SUVmax.eq = 4.7) was also observed, suggesting endometrial malignancy or physiological uptake (**Figure 1D**).

**Figure 2:**
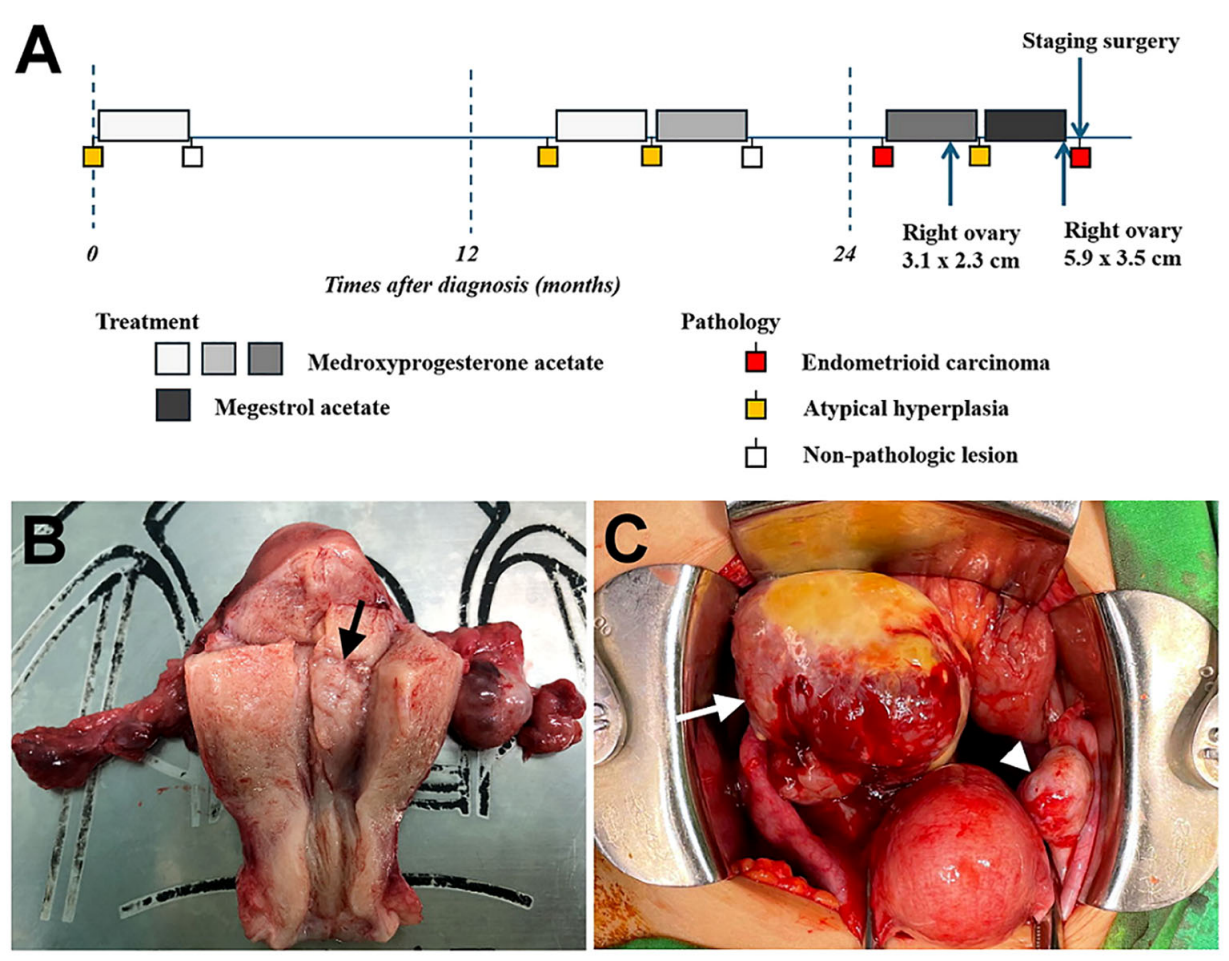
**(A)** Timeline of the patient’s treatment process. **(B)** Endometrial lesion (arrow) measuring 1.3 cm observed in the endometrial cavity of uterus. **(C)** Enlarged right ovary (arrow) and normal ovary (arrowhead) noted during operation.

**Figure 3:**
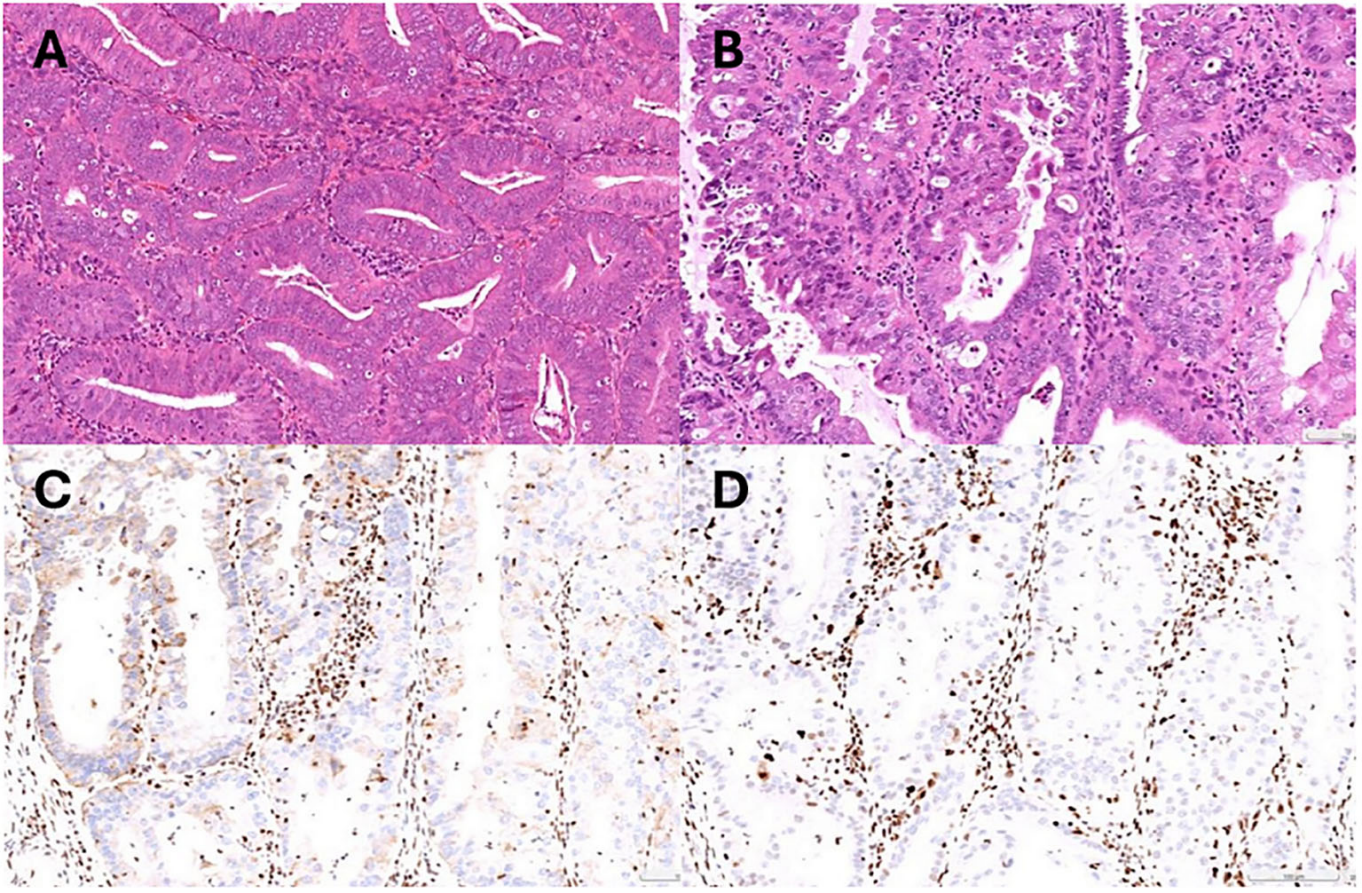
**(A)** Pathology of endometrial biopsy confirmed atypical hyperplasia at initial diagnosis. **(B)** Representative microscopic photo demonstrating grade 2 endometrioid carcinoma (Hematoxylin and eosin stain). **(C)** Immunohistochemical stain showing MSH2 loss. **(D)** Immunohistochemical stain showing MSH6 loss.

A comprehensive staging surgery was performed (**Figures 2B, C**). Histologic examination reported endometrial endometrioid carcinoma, grade 2, with tumor invading more than half of the myometrium and testing positive for lymph-vascular space invasion. Right ovarian endometrioid carcinoma was consistent with metastasis from uterine cancer. The patient’s treatment timeline is shown in **Figure 2A**, illustrating the rapid progression of her disease despite progestin treatment. Immunohistochemically, tumor cells were positive for PAX8, ER, and PMS2, whereas MSH6 and MSH2 were negative (**Figure 3**). Microsatellite instability (MSI) testing by Next-Generation Sequencing (NGS) also confirmed to be MSI-high, with genetic variants at MSH2 (R711*, splice site 2006-2A>G) and MSH6 (F1088fs*2), concordant with immunohistochemistry (IHC) stain. The final diagnosis was endometrial endometrioid carcinoma, FIGO 2023 stage IIIA1.

The patient recovered well postoperatively and has been undergoing adjuvant treatment with chemotherapy and immunotherapy. Tumor markers returned to the normal range since the third cycle of adjuvant therapy. The patient continues to be closely monitored, and there has been no evidence of recurrence at the 12-month follow-up.

## 3 Discussion

Lynch syndrome is a hereditary disease caused by germline mutations in mismatch repair (MMR) genes, significantly increasing the lifetime risk of colorectal, endometrial, and ovarian cancers in women. The risk of developing EC in LS patients is 40-60%, which is much higher than in the general population (9). Characteristics of EC in LS include a younger age of onset, well-differentiated features, and diagnosis at an early stage. However, no agreement exists on whether LS patients are appropriate candidates for fertility preservation. One of the concerns is that LS also increases the lifetime risk of other malignancies. 13.7% of patients have synchronous cancers at the initial diagnosis, with the highest proportion being synchronous colon and endometrial cancers (8). Different pathogenic MMR genes carry varying risks of developing cancer. The cumulative incidences of colorectal cancer up to the age of 75 were 46%, 43%, and 15% for MLH1, MSH2, and MSH6 mutation carriers, respectively. For EC, the incidences are higher in MSH2 and MSH6, reaching 57% and 46%, respectively (10). Ovarian cancer ranks as the third most common cancer in women with LS (7). Other less common cancer types, such as gastric, pancreatic, biliary tract, brain, and urinary tract cancers, have also been reported in association with LS (10). Given these complexities, comprehensive assessments are imperative before Lynch-associated EC patients opt for fertility-sparing treatment. Surveillance strategies should be tailored based on age and the specific pathogenic MMR genes involved. The occurrence of synchronous or metachronous cancers may influence the decision regarding fertility-sparing treatment.

The classification of endometrial cancer has evolved from histological to molecular subtypes following the introduction of four molecular categories by The Cancer Genome Atlas (TCGA) Research Network in 2013. This new classification system identifies four categories with distinct clinical and molecular features: POLE-mutated, MMR-deficient (MMRd), p53-abnormal, and no specific molecular profile (NSMP). Among patients with low-grade endometrial cancer, 60% are classified as NSMP and 25% as MMRd (11). Some reports suggested that patients with a defect in MMR genes experienced inferior treatment outcomes when undergoing progestin therapy (12, 13). Zakhour et al. and Dagher et al. found that none of the patients with abnormal IHC for MMR proteins showed resolution of hyperplasia or malignancy through conservative treatment (12, 13). Chung et al. observed higher upstaging rates in the final pathological reports of patients with MMRd. However, there was no significant difference in the recurrence rate after achieving CR compared to patients with MMR-proficient (MMRp) (14). In contrast, Raffone et al. reported that the MMRd was correlated with a higher recurrence rate (15). Their study also highlighted that combining hysteroscopic resection with progestin treatment resulted in a higher response rate compared to previous studies, potentially facilitating attempts at pregnancy (15).

**Table 1** summarizes the outcomes of LS and MMRd patients who received fertility-sparing treatment in 7 studies and ours (12–18). The CR or PR rate was lower than that of the general population, with recurrence rates ranging from 25% to 100%. There were 11 cases of recurrence, with the time from CR to relapse ranging from 3 to 39 months. Our patient’s first time to recurrence was at 12 months. Most of the relapse patients underwent hysterectomy. Only one patient successfully achieved childbirth through surrogacy. Notably, one patient diagnosed with LS experienced progestin treatment failure, leading to the identification of stage IA grade 1 EC with synchronous stage IC1 unilateral ovarian clear cell carcinoma (12). This finding is consistent with concerns regarding synchronous tumors in LS. Nevertheless, to date, most studies are retrospective and constrained by small sample sizes, lacking conclusive data on the effectiveness of fertility-sparing treatment in early-stage EC or AEH among LS or MMRd patients.

**Table 1:** Outcomes of conservative treatment for EC and AEH in patients with LS or MMRd.

Study	Year	Age (years)	No	Germline test	Pathology of initial biopsy*	Final pathology*	CR+PR rate	Recurrence after CR	Hysterectomy	Follow-up (months)	Pregnancy rate
Zakhour (13)	2017	42 (33–55)	6	LS (3)	EC 1AG1 (3)	EC 1AG1 (1) EC 1AG2 (1) EC 1BG2 (1)	0 (0/6)	NA	100% (6/6)	77 (15–201)	NA
				MMRd (3)	AEH (1) EC 1AG1 (2)	AEH (1) EC 1AG1 (1) EC 1AG2 (1)					
Falcone (16)	2019	36 (28–39)	7	LS (2)	EC 1AG1 (2)		71.4% (5/7)	20%	28.6% (2/7)	73.7 (24–125)	NA
				MMRd (5)	EC 1AG1 (4) EC 1AG2 (1)	EC 1AG1 (1) EC 1AG3 (1)					
Chung (14)	2021	33 (26–40)	9	NA	EC 1AG1 (7) EC 1AG2 (2)	EC 1AG1 (1) EC 1BG1 (1) EC 1AG3 (1) EC 1A, carcinosarcoma (1)	44.4% (4/9)	25%	44.4% (4/9)	66.7 (13.2–163.5)	NA
Raffone (15)	2021	29 (31–43)	6	NA	AEH (3) EC (3)	NA	66.7% (4/6)	100%	NA	54.8 (12-89.2)	NA
Puechl (17)	2021	72 (52–87)	6	NA	AEH (2) EC (4)	EC (2)	66.7% (4/6)	NA	33.3% (2/6)	28.8 (3.3–141.5)	NA
Catena (18)	2022	36 (31–43)	6	LS (3)	AEH (3)	AEH (2) EC 1AG1 (1)	83.3% (5/6)	100%	100% (6/6)	20 (12–42)	0%
				MMRd (3)	AEH (1) EC 1AG1 (2)	AEH (1) EC 1AG1 (2)					
Dagher (12)	2023	41 (34–48)	3	LS (1)	AEH (1)	EC 1AG1 & OVCA 1C1 (1)	0 (0/3)	NA	100% (3/3)	88.2 (87–100)	33.3% (1, surrogacy)
				MMRd (2)	AEH (1) EC 1AG1 (1)	EC 1AG1 (1) EC 3C, mixed G2 & clear cell (1)					
Our case	2023	39	1	LS	AEH	EC 3AG2^†^	100%	100%	100%	44	NA

Notes: Data are presented as mean (range), and valves in parenthesis are numbers unless otherwise specified.

Abbreviations: AEH, atypical endometrial hyperplasia; EC, endometrial cancer; LS, Lynch syndrome; MMRd, mismatch repair deficiency; CR, complete response; PR, partial response; SD, stable disease; PD, progressive disease; G1; grade 1; G2, grade 2; G3, grade 3; OVCA, ovarian cancer; NA, not available.

*FIGO 2009 staging system

^†^ FIGO 2023 staging system

Treatment outcomes for women with early-stage EC or AEH undergoing conservative therapy are generally favorable. Previous studies have shown acceptable retreatment rates with hormone therapy, with CR rates ranging from 82% to 88% for the first retreatment and 70% for the second retreatment (19, 20). Regarding retreatment after recurrence, no studies have investigated MMRd or LS patients, although the recurrence rate was high among these patients, ranging from 25% to 100% (**Table 1**). In our presented case, the patient’s AEH was successfully treated with fertility-sparing therapy and achieved CR initially. The pathology confirmed EC at the second recurrence, but a third round of fertility-sparing treatment was initiated due to the patient’s strong desire to preserve fertility. Subsequent biopsy results indicated only a partial response and a new adnexal mass was identified, later confirmed to be EC with ovarian metastasis after staging surgery (**Figure 2A**). This case highlights the importance of vigilance in monitoring LS patients during re-treatment, as demonstrated by the rapid progression despite progestin treatment. Close surveillance and consideration of surgical treatment strategies may be necessary in such cases.

## 4 Conclusion

Our patient responded well initially to progestin treatment but experienced two recurrences in a short interval, potentially linked to the genetic defect associated with Lynch syndrome. Limited research on retreatment in this group warrants cautious monitoring. Despite fertility-sparing therapy showing promising response, vigilance and consideration of surgical intervention are crucial post-relapse. Despite fertility-sparing therapy in early-stage EC and AEH showing promising response, vigilance and consideration of surgical intervention are crucial post-relapse.

## Data Availability

The datasets presented in this study can be found in online repositories. The names of the repository/repositories and accession number(s) can be found in the supplementary material.
